# Poverty, Firearm Deaths, and Racial Disparities: Repercussions of the First Year of the COVID-19 Pandemic Across the United States

**DOI:** 10.7759/cureus.111167

**Published:** 2026-06-19

**Authors:** Karan Varshney, Minh Giao Chu

**Affiliations:** 1 Department of Medicine, Peninsula Health, Frankston, AUS; 2 Department of Surgery, Albury Wodonga Health, Wodonga, AUS

**Keywords:** covid-19, firearm injury, public health and safety, race inequities, urban poverty

## Abstract

Background: The COVID-19 pandemic has had devastating direct impacts on health and has also contributed indirectly to a wide array of socioeconomic issues. In the United States (US), these impacts have been shown to be particularly pronounced among racial and ethnic minorities. This study aimed to assess the impacts of the first year of the COVID-19 pandemic on poverty rates and firearm deaths, with a specific focus on the impacts amongst racial minorities in the US.

Methods: This ecological study analyzed US state-level data on poverty rates and firearm death rates for the years 2019 and 2020 to assess changes in firearm deaths and poverty rates during the first year of the COVID-19 pandemic. Changes in poverty rates and changes in firearm death rates by state were determined, and regression analysis was conducted to evaluate the relationship between these rates and racial minority population rates.

Results: There was a mean increase of 12.15% in poverty rates and a mean increase of 12.96% in firearm death rates, with an increased proportion of the Black/African American population having a statistical relationship with changes in firearm death rates (β = 0.310; p = 0.032).

Conclusions: Our study highlights that certain communities may have been disproportionately impacted by the COVID-19 pandemic on numerous, previously understudied parameters. Further interventions and studies are needed to better tailor support for vulnerable communities.

## Introduction

The COVID-19 pandemic has had devastating consequences on the United States (US) population. In addition to the direct public health impacts, there have also been effects on employment and finances, leading to economic and social stress among many [1,2]. A cohort study of 1,392 working-age adults in the US found that early pandemic income or job loss was associated with a 9-11% increase in psychological distress, persisting for up to two years, even after adjusting for baseline distress, financial status, and sociodemographics [3].

Furthermore, due to factors relating to systemic racism and inequitable social determinants of health, racial minorities have been disproportionately affected by COVID-19 in numerous ways [4,5]. COVID-19 infection and mortality rates have been significantly higher among African American, Hispanic, and Indigenous communities compared to their Caucasian counterparts. This disparity has been attributed to multiple factors, including the overrepresentation of people of color in essential occupations with increased risk of exposure [6]. Yet, during the early phase of the COVID-19 pandemic, it was noted that job losses also disproportionately affected Hispanic, Black, and Asian workers compared to Caucasian workers, a disparity driven by differences in industry and occupational exposure, as well as lower rates of remote work capability among minority groups [7,8].

The social upheaval and uncertainty of the COVID-19 pandemic coincided with a sharp increase in firearm purchases across the US [9,10]. This surge appears to have been fueled by layered psychological stressors, including grief, isolation, and anxiety, compounded by widespread economic hardship and deep-rooted racial and health inequities [11]. Notably, this escalation in firearm acquisition occurred alongside a measurable rise in firearm violence: during the pandemic’s first year, the US recorded over 8,000 additional firearm incidents, a 15% increase, along with a 34% jump in gun-related injuries and a 28% rise in fatalities [12]. States such as Minnesota and New York saw particularly acute spikes, and nationally, the firearm homicide rate climbed from 4.7 to 6.4 per 100,000 [12]. However, there remains a lack of causal evidence to fully explain the observed rise in firearm purchases and gun-related deaths during the COVID-19 pandemic.

There is currently a need to better quantify the immediate impacts of COVID-19 on social and economic measures relating to health and to determine the extent to which vulnerable racial minorities are disproportionately impacted. Therefore, the objectives of our analysis were as follows: first, we sought to analyze the changes in poverty rates and firearm death rates by state during the first year of the COVID-19 pandemic. It also aimed to evaluate whether these changes were associated with one another. Lastly, we aimed to assess if these rates were associated with the proportion of Black/African American and Hispanic/Latino populations within states.

## Materials and methods

This ecological study analyzed US state-level data on poverty rates and firearm death rates for the years 2019 and 2020 to assess changes in firearm deaths and poverty rates during the first year of the COVID-19 pandemic, utilizing bivariate and regression models to evaluate for associations with socioeconomic and racial demographic factors [13-15].

All data utilized in this study are publicly available and do not include any identifiable data on any individuals; hence, ethics board approval was not required for this study. There was no need to address missing data, as complete data from these publicly available datasets were utilized.

To assess whether there were changes in rates during the COVID-19 pandemic, US state-level data on poverty and firearm death rates for 2019 and 2020 were obtained from the Centers for Disease Control and Prevention's (CDC) National Center for Health Statistics [13-15]. Data were included for 50 states (excluding the District of Columbia due to insufficient data). Data were collated on Microsoft Excel (Microsoft Corporation, Redmond, Washington). The percent change between 2019 and 2020 was then calculated for these respective rates using the following formula: Percent change = ((2020 value - 2019 value) / 2019 value) * 100. After obtaining percent-change values for poverty and firearm rates, the data were visualized by state in a graph format; ranges, means, medians, and standard deviations were also reported. Next, data on the proportions of Black/African American and Hispanic/Latino in total state populations were obtained from the US Population Census Survey [15]. These data were also collated in Microsoft Excel and subsequently depicted visually as graphs. These minority groups were specifically chosen, as it has been demonstrated previously that they have been disproportionately impacted by the COVID-19 pandemic and have higher death rates [16]. Graphs were created using PRISM GraphPad version 9.5.1 (GraphPad Software, San Diego, California).

Bivariate regression analysis was next conducted to assess if there was a relationship between the variables. The relation between Hispanic/Latino and Black/African American and changes in poverty rate were each compared with changes in firearm death rates; this was similarly done for each race variable and changes in the poverty rate. Finally, multivariable regression analysis was conducted using two separate models, with changes in the poverty rate as the outcome variable in one model and changes in firearm death rates as the outcome variable in the other model. Variables were automatically excluded if there was a variable inflation factor greater than eight. Standardized beta coefficients, 95% confidence intervals, p-values, and t-statistics were all reported. Statistical analysis was conducted using IBM SPSS Statistics for Windows, Version 28 (Released 2021; IBM Corp., Armonk, New York).

## Results

Between 2019 and 2020, poverty rates increased in 36 states, decreased in 12, and remained unchanged in two. The mean increase in poverty rates for states was 12.15% (SD = 19.15). The largest increases were seen in New Hampshire (67.57), followed by Delaware (61.54), and Minnesota (49.12). The largest decreases occurred in Maine (-23.08) and Illinois (-13.98).

**Figure 1:**
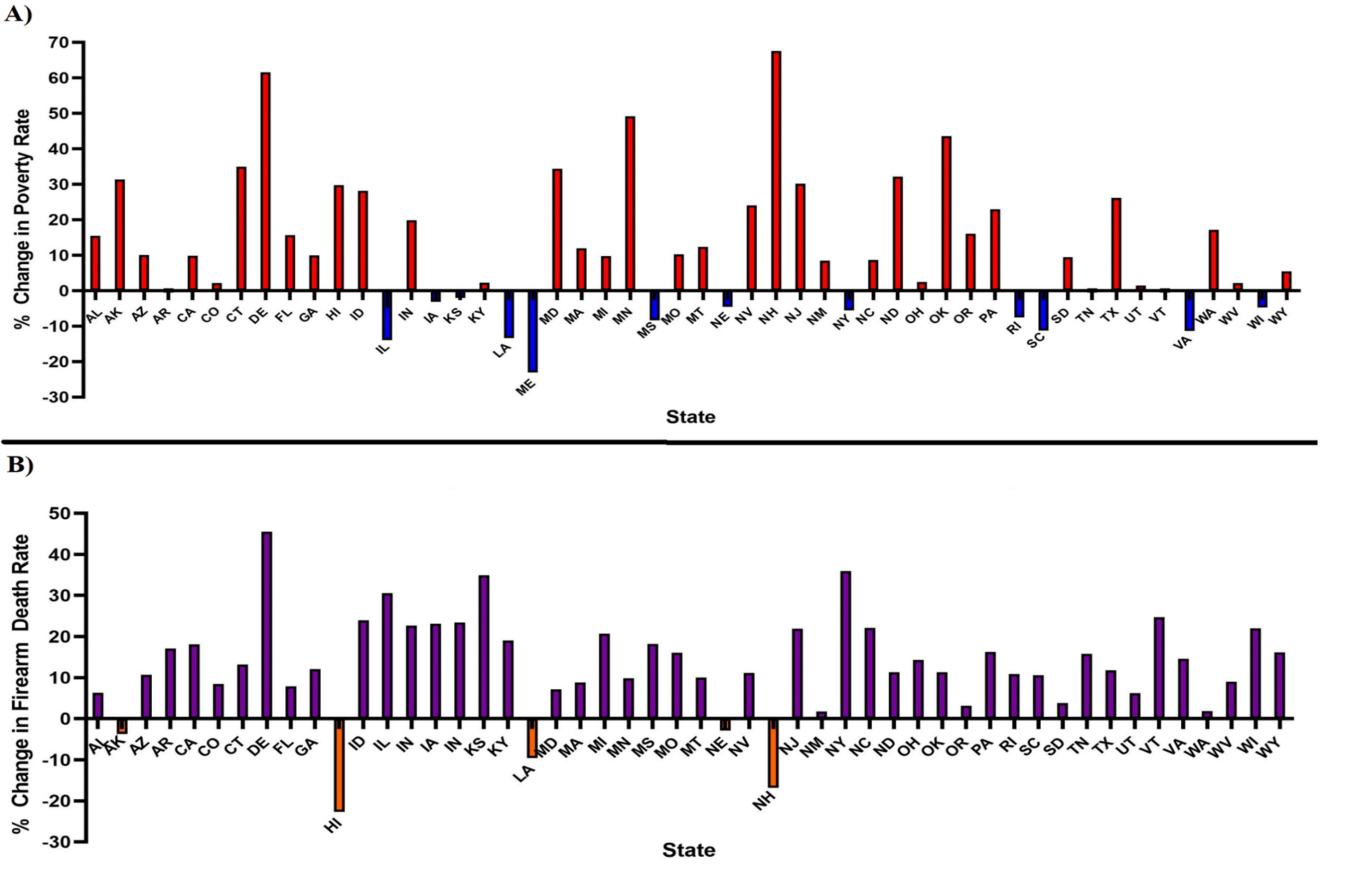
Percent change by state, between 2019 and 2020, of poverty rates (A), and firearm deaths (B)

Regarding firearm death rates, there was a percent increase between 2019 and 2020 across 45 states and a decrease in five states. The mean increase in firearm death rates was 12.96% (SD = 12.17). Increases were largest in Delaware (45.46), followed by New York (35.90), and Kentucky (34.90). The highest decreases occurred in Hawaii (-22.73) and New Hampshire (-16.82). Percent changes by state are depicted in Figure 1.

**Figure 2:**
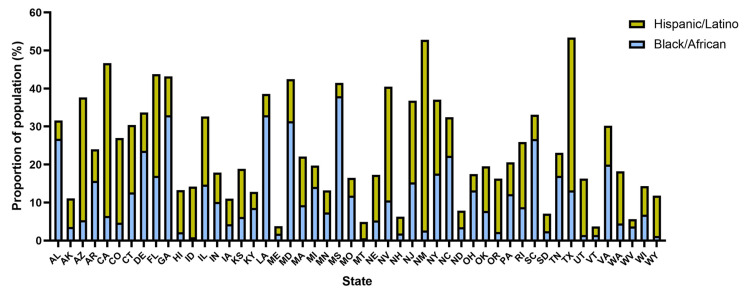
Racial minorities (Hispanic/Latino and Black/African American) proportions of total population by state

Figure 2 depicts the Hispanic/Latino and Black/African American populations as a proportion of the total state population. For bivariate regression with changes in poverty rates as the outcome variable, there was no relationship with the Hispanic/Latino population (β = 0.068; p = 0.640) and no relationship with the Black/African American population (β = -0.116; p = 0.422). With poverty rate changes as the outcome, there was no relationship with the Hispanic/Latino population (β = -0.020; p = 0.311), though there was a statistical relationship with the Black/African American population (β = 0.322; p = 0.022). Bivariate analysis showed no statistical relationship between changes in poverty rates and changes in firearm deaths (β = -0.160; p = 0.268).

The results of the multivariable models are shown in Table 1. For multivariable regression with poverty rate changes as the outcome, there was no statistical relationship with any of the three predictor variables. The change in poverty rate was therefore not associated with the change in firearm violence rate (β = -0.137; p = 0.374), proportion of the population that is Black/African American (β = -0.065; p = 0.674) or proportion of the population that is Hispanic/Latino (β = 0.058; p = 0.695). For the second model with changes in firearm death rate as the outcome, there was only a statistical relationship with the proportion of the population that is Black/African American (β = 0.310; p = 0.032); there was no statistical relationship with changes in firearm death rates and changes in poverty rate (β = -0.125; p = 0.374) or the proportion of the population that is Hispanic/Latino (β = 0.023; p = 0.163).

**Table 1:** Results of multivariable regression models evaluating relationships between poverty, gun violence, and race* * Variance inflation factor for all variables in both models was less than eight.

Predictor variable	Standardized β-value	95% CI lower bound	95% CI upper bound	p-value	t-statistic
Model 1 – Outcome variable: Change in poverty rate (2019–2020)					
Change in firearm violence rate (2019–2020)	-0.137	-0.701	0.269	0.374	-0.897
Black/African American population (proportion of total)	-0.065	-0.745	0.486	0.674	-0.424
Hispanic/Latino population (proportion of total)	0.058	-0.428	0.636	0.695	0.395
Model 2 – Outcome Variable: Change in firearm death rate (2019–2020)					
Change in poverty rate (2019–2020)	-0.125	-0.258	0.099	0.374	-0.897
Black/African American population (proportion of total)	0.310	0.036	0.747	0.032	2.217
Hispanic/Latino population (proportion of total)	0.023	-0.297	0.349	0.871	0.163

## Discussion

In this study, we have quantified some of the immediate social impacts of COVID-19. Both poverty rates and firearm death rates have seen increases across states. Our analysis has highlighted that, while these rates have both increased, they are not necessarily linked and intertwined. This demonstrates that social and economic recovery efforts from the impacts of the pandemic need to be implemented in differing ways depending on the unique challenges faced by a particular state, and allocation of funding should match state needs. For example, while in Illinois, the poverty rate decreased by nearly 15% between 2019 and 2020, and the firearm deaths rose by more than 30% in that time period, New Hampshire instead had a nearly 68% increase in poverty rates but a nearly 17% decline in gun violence; states such as Delaware saw a sharp rise in both rates. These respective states will, hence, have differing needs.

Our analysis has described the complexities of emergent social issues due to COVID-19 among racial minorities. In many ways, the direct impacts of the pandemic on Hispanic/Latino and Black/African American populations have been disproportionately large and hence indicate a need for services and programs implementation to reduce health inequities [16]. However, for indirect impacts, the relationship is not as clear. It has been demonstrated that deaths by firearms are linked to an increased proportion of Black/African Americans in the total state population. Possible reasons for this are the increased trauma that this group experienced during the pandemic due to issues such as police brutality and awareness of the pandemic’s particularly large impact on Black populations as a result of deep-rooted systemic racism [17,18]. It is recommended that programs be developed to understand and address the unique traumas faced by Black/African American populations in areas where gun violence has had the largest increases.

Building on this, a mixed-methods study by Patton et al. (2022) among Black and Latinx New York Housing Authority residents during the COVID-19 pandemic highlights how communities most impacted by firearm violence are actively calling for public health-oriented solutions, combining trauma-informed mental health care, youth programming, and trusted peer-led violence interruption strategies that foster relational safety and long-term resilience [19]. It is critical to emphasize that race itself is not the explanatory factor for these findings, and it is important to recognize the significant role of structural inequalities associated with race. Therefore, factors such as systemic racism, trauma, hate crimes, and discrimination are likely to contribute to inequities - it is of paramount importance to address these systemic factors to support impacted populations. Multiple national initiatives have emerged to address firearm violence in Black American communities, including Community Violence Intervention programs that employ trusted community members to mediate conflict and connect high-risk individuals with services. Complementary efforts include firearm safety education (e.g., Black Guns Matter), public health advocacy by groups like Giffords and Everytown, and broader policy measures such as safe storage laws [20]. However, current intervention efforts may continue to fall short in addressing the deep-rooted structural and socioeconomic inequities that have arisen from a long history of racial discrimination - an enduring gap that demands recognition and sustained, systemic reform.

This work has important limitations to consider. As we performed an ecological analysis, there was limited capability for us to analyze the unique factors in states, and racial minority populations, that may have driven changes. For ecological studies such as ours, it is critical to avoid the ecological fallacy, where population-level trends are incorrectly applied at the individual or small group level. Additionally, these findings are only able to show associations at a population level and cannot establish causality. Therefore, we recommend that future research be conducted to understand the social and economic impacts of COVID-19, both nationally and locally in each state, and specifically to assess how minority groups are uniquely impacted. Furthermore, it is plausible that data may not have accounted for impacts faced by undocumented people; important considerations may have been missed as a result. In addition, we were not able to assess the impacts of the COVID-19 pandemic on mass shooting events, which have been shown to be linked to a wide array of complex social phenomena, and this should be analyzed further in future studies [21,22]. The lack of addressing confounding variables, as well as the study being restricted to only two years, are other important limitations to consider. Factors that may have impacted the findings but were not accounted for include unemployment rates, education levels, extent of urbanization, degree of firearm ownership, policing practices, mental health access, political policies, and the severity of COVID-19 at the state level. Regardless of the limitations, this work has the impact to shape public policy that can improve population health and to continue to understand the social factors that impact vulnerable communities.

## Conclusions

Our study highlights that the social impacts of the COVID-19 pandemic have been uneven and complex, with rising firearm deaths and changing poverty rates revealing deep inequalities linked to race and history. These findings suggest a need for more research to understand how systemic racism, economic stress, and health crises interact and impact racial minorities; these findings highlight the importance of supporting ongoing research, initiatives, and programs that can provide community support.
